# Cost-effectiveness of Algorithms for Confirmation Test of Human African Trypanosomiasis

**DOI:** 10.3201/eid1310.060358

**Published:** 2007-10

**Authors:** Pascal Lutumba, Filip Meheus, Jo Robays, Constantin Miaka, Victor Kande, Philippe Büscher, Bruno Dujardin, Marleen Boelaert

**Affiliations:** * Programmed National de Lutte contre la Trypanosomiase Humaine Africaine, Kinshasa, Democratic Republic of Congo; † Institute of Tropical Medicine, Antwerp, Belgium; ‡ Royal Tropical Institute, Amsterdam, the Netherlands; § Ecole de Santé Publique, Université Libre de Bruxelles, Brussels, Belgium

**Keywords:** Human trypanosomiasis, diagnostic accuracy, Africa, sensitivity, specificity, Trypanosoma brucei gambiense, screening, algorithm, research

## Abstract

The control of *Trypanosoma brucei gambiense* human African trypanosomiasis (HAT) is compromised by low sensitivity of the routinely used parasitologic confirmation tests. More sensitive alternatives, such as mini-anion exchange centrifugation technique (mAECT) or capillary tube centrifugation (CTC), are more expensive. We used formal decision analysis to assess the cost-effectiveness of alternative HAT confirmation algorithms in terms of cost per life saved. The effectiveness of the standard method, a combination of lymph node puncture (LNP), fresh blood examination (FBE), and thick blood film (TBF), was 36.8%; the LNP-FBE-CTC-mAECT sequence reached almost 80%. The cost per person examined ranged from €1.56 for LNP-FBE-TBF to €2.99 for LNP-TBF-CTC-mAECT-CATT (card agglutination test for trypanosomiasis) titration. LNP-TBF-CTC-mAECT was the most cost-effective in terms of cost per life saved. HAT confirmation algorithms that incorporate concentration techniques are more effective and efficient than the algorithms that are currently and routinely used by several *T.b. gambiense* control programs.

## Abstract 2

Algorithms that incorporate concentration techniques are more effective and efficient than the currently used algorithms.

Human African trypanosomiasis (HAT) is a parasitic disease that affects 36 countries in sub-Saharan Africa. The most recent World Health Organization (WHO) prevalence estimates are 50,000–70,000 cases worldwide, based on a total number of 17,500 new HAT cases per year worldwide (*1*). *Trypanosoma brucei gambiense* HAT control activities are based principally on the active detection of cases by population screening and subsequent treatment of infected patients. Because of the relative toxicity of HAT drugs, a correct diagnosis is essential before the treatment can begin (*2*).

The specificity of the card agglutination test for trypanosomiasis (CATT) used in screening is not 100% accurate, so HAT control programs use a variable sequence of parasitologic tests as confirmation tests. In the Democratic Republic of Congo (DRC), this sequence, called the standard algorithm, comprises lymph node puncture (LNP), followed by fresh blood examination (FBE) and thick blood film (TBF). Several authors have reported on the low sensitivity levels of HAT confirmation tests (*3*,*4*). Paquet et al. reported that mobile HAT screening teams in Uganda detected only 39% of all HAT cases (*5*). HAT cases missed by population screening will later be diagnosed by fixed health services operating in the same areas as the mobile teams, but almost invariably not until the late stages of the disease. HAT confirmation is more straightforward (*6*). Late-stage detection is problematic because it carries a much poorer prognosis for the patient and forgoes the principal public health objective of HAT control, which is a rapid reduction in transmission. \Technical solutions to increase the sensitivity of screening algorithms do exist. Several concentration tests have been proposed, notably the mini-anion exchange centrifugation technique (mAECT) (*7*), capillary tube centrifugation (CTC) (*8*), and the quantitative buffy coat (QBC) (*9*). A recent study of 436 case-patients conducted in Kwamouth, DRC, showed that 154 had parasitologic-confirmed HAT cases. Although good sensitivity was reported, and mAECT and CTC were relatively simple to implement, it is not economically feasible to use these innovative tools in the field.

Other authors have proposed recourse to serology using higher cut-offs of the CATT test to increase specificity (referred to as CATT titration, in contrast with CATT whole blood, which is the test used in the first screening step) as part of the algorithm for use in screening, or as a test to decide whether to treat. The present study is an analysis of the cost-effectiveness and value for HAT control policy of different HAT confirmation algorithms, including serologic algorithms.

## Methods

A decision-analysis model was used to estimate the effectiveness and cost-effectiveness of a number of HAT screening-treatment algorithms; different HAT confirmation test sequences were compared, including concentration techniques and CATT titration. Decision analysis is a method that quantifies the value of several alternative options in a complex choice. This technique requires the construction of a decision tree, which shows detailed options, estimation of the node probabilities, and an evaluation of the economic or public health consequences of each option (John M, unpub. data) (*10*,*11*). A health service perspective was taken for this analysis, which includes all screening and treatment costs generated by the choice of HAT test algorithms.

### Decision Tree

Fourteen HAT control experts were asked to identify all relevant test sequences that would confirm HAT in a given person (i.e., positive in the initial screening test, CATT). The availability of these tests was an important factor in the expert poll. At the time of this study, QBC was no longer manufactured, and for this reason, it was not included in our analysis. We analyzed 7 algorithms as shown in Table 1. All algorithms imply that only parasitologic positives will be put on stage-dependent treatment except for the fourth group (serologic algorithm), which implies stage-dependent treatment of persons who are negative for parasites but positive by CATT titration.

**Table 1:** Screening algorithms for human African trypanosomiasis*

Label	Algorithm	Abbreviation	
Algorithm 1 or standard	Lymph node puncture, fresh blood examination, and thick blood film	LNP-FBE-TBF	
Algorithm 2	Lymph node puncture and capillary tube centrifugation	LNP-CTC	
Algorithm 3	Lymph node puncture, CATT titration, capillary tube centrifugation, and mini-anion exchange centrifugation technique	LNP-CATT titration-CTC-mAECT	
Algorithm 4	Lymph node puncture, capillary tube centrifugation, and mini-anion exchange technique	LNP-CTC-mAECT	
Algorithm 5	Lymph node puncture, thick blood film, capillary tube centrifugation, and mini-anion exchange technique	LNP-TBF-CTC-mAECT
Algorithm 6	Lymph node puncture, capillary tube centrifugation, and CATT titration	LNP-CTC-CATT titration
Algorithm 7	Lymph node puncture, thick blood film, capillary tube centrifugation, mini-anion exchange technique, and CATT titration	LNP-TBF-CTC-mAECT-CATT titration

*CATT, card agglutination test for trypanosomiasis; CATT titration, CATT titration at end-titer 8.

**Figure 1:**
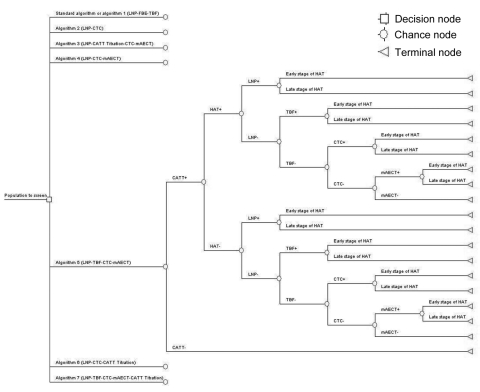
Decision tree comparing algorithms used to analyze human African trypanosomiasis (HAT). LNP, lymph node puncture; FBE, fresh blood examination; TBF, thick blood film; CTC, capillary tube centrifugation; CATT, card agglutination test for trypanosomiasis; CATT titration, CATT titration at end-titer 8; mAECT, mini-anion-exchange centrifugation technique.

We constructed a decision tree comparing the algorithms mentioned above (Figure 1); the entry point is a person who participates in HAT population screening conducted by a mobile team. There are 4 possible outcomes for such a person: a true HAT case-patient is treated, a true HAT case-patient remains untreated, a non-HAT case-patient receives HAT treatment, or a non-HAT case-patient remains untreated. These 4 outcomes were evaluated in terms of lives saved.

### Probabilities

Table 2 shows the probabilities used in this decision analysis. The baseline values were generated in a study carried out in Kwamouth between February and May 2004 (*4*) or were retrieved from the literature. In the baseline scenario, we assumed HAT prevalence in the community to be 1%. This value is a limit used by HAT-control programs to distinguish between severe and nonsevere HAT foci. A literature search was performed by using the Medline database to find information reported between 1950 and 2005 to identify baseline values of parameters with a plausible range. A sensitivity analysis was performed to test consistency of our conclusions over the range of plausible values.

**Table 2:** Probabilities used in baseline scenario and plausible range*

Characteristic	Baseline value, %	Reference	Plausible range, %	Reference
HAT prevalence	1.0	Annual reports PNLTHA (1995–2002)	0.5–5.0	Annual reports PNLTHA (1995-2002)
LNP sensitivity	18.8	(*20*)	18.8–58.6	(*3*,*4*)
LNP specificity	100.0	By convention	NA	
FBE sensitivity	3.9	(*4*)	3.9–22.4	(*3*,*4*)
FBE specificity	100.0	By convention	NA	
TBF sensitivity	27.3	(*4*)	27.3–34.5	(*3*,*4*)
TBF specificity	100.0	By convention	NA	
CTC sensitivity	56.5	(*4*)	29.0–73.0	(3,7)
CTC specificity	100.0	By convention	NA	
mAECT sensitivity	75.3	(*4*)	43.0–88.0	(3,7)
mAECT specificity	100.0	By convention	NA	
CATT whole blood sensitivity	90.4	(*23*)	68.8–99.2	(*21*–*24*); John M (unpub. data)
CATT whole blood specificity	96.5	(*23*)	83.5–98.4	(*21*,*23*,*24*)
CATT titration sensitivity†	78.8	(*4*)	78.8–100.0	In absence of data in literature, we considered the maximum of 100%
CATT titration specificity†	59.0	(*4*)	59.0–100.0	In absence of data in literature, we considered the maximum of 100%
Pentamidine efficacy	98.0	‡	98.0–99.0	(*25*)
Melarsoprol efficacy	90.0	‡	70.0–96.3	(*25*,*26*)
Latrogenic mortality of pentamidine	0.1	‡	0.1–0.7	(*25*)
Latrogenic mortality of melarsoprol	2.0		2.0–7.0	(*25*–*27*)

*HAT, human African trypanosomiasis; PNLTHA, Programme National de lutte contre la Trypanosomiase Humaine Africaine; LNP, lymph node puncture; NA, not applicable; FBE, fresh blood examination; TBF, thick blood film; CTC, capillary tube centrifugation; mAECT, mini-anion exchange centrifugation technique; CATT, card agglutination test for trypanosomiasis. †Conditional values to CATT whole blood positive. The plausible range included the value at different end-titers (4, 16, 32). ‡Data extracted from PNLTHA/RDC annual report 1999.

### Effectiveness

The effectiveness of each HAT screening-treatment algorithm was estimated, taking into account all steps, including screening, confirmation, and treatment. The results were quantified in terms of the number of lives saved (confirmed case, treated, and cured) by each algorithm. HAT treatment decision depends on disease staging (*12*). A diagnostic algorithm can theoretically generate 4 different outcomes: true positive, false positive, true negative, and false negative.

Effectiveness values were assigned to each of the alternatives. A true HAT case-patient who is treated is equal to 0.9 lives saved because the efficacy of HAT treatment is estimated at 90%, according to data from the Programme National de Lutte contre la Trypanosomiase Humaine Africaine (PNLTHA) between 1996 and 2002. For a few persons, treatment of non-HAT case-patients with toxic drugs will lead to iatrogenic death. Therefore, an effectiveness value was assigned to this endpoint of –0.001 lives saved for first-stage drugs and –0.020 lives saved for second-stage drugs. A non-HAT case-patient and a true HAT case-patient who was untreated were each assigned an effectiveness value of 0 lives saved. Effectiveness was expressed as the percentage of HAT deaths averted by a strategy. To obtain the actual number of lives saved by the strategy, this value must be multiplied by HAT prevalence.

### Costs

While assessing the different algorithms, we distinguished between 3 steps in the process: 1) screening, 2) confirmation, and 3) treatment. Using the ingredient approach, we estimated the cost of screening by the mobile team for HAT on the basis of observations made in 2003. A detailed overview of cost items is given in Table 3. The cost of the screening step includes all equipment required to set up a mobile team: vehicles, depreciation, operating costs, and CATT reagents.

**Table 3:** Annual cost of the operations of a mobile team for human African trypanosomiasis active case finding, Democratic Republic of Congo, 2003

Input category	Annual cost, €†	% Total cost
Capital		
Vehicles	5,125.00	11
Medical and lab equipment	2,760.00	6
Training	671.66	1
Other equipment	1,416.75	3
Subtotal	9,973.41	21
Recurrent		
Personnel	11,520.00	25
Medical and lab supply	14,798.52	32
Essential drugs, not HAT	2,100.00	4
Stationary	2,842.36	6
Vehicles, operation and maintenance	5,200.00	11
Other operating input	300.00	1
Subtotal	36,760.88	79
Total	46,734.29	100

*US $1 = €0.86, May 2003.

For the second step (HAT confirmation), each confirmation test was assessed in terms of resources consumed, equipment depreciation, and time taken by a mobile team to realize each test. Data on costs and time were collected during a validation study conducted in Kwamouth in 2004 (*4*). The time for each diagnostic test was estimated by using a stopwatch for a sample of 50 procedures, and the results were measured in terms of minutes elapsed. The cost of LNP has been estimated as €0.19, FBE at €0.21, TBF at €0.54, CTC at €0.76, and mAECT at €2.82 (*4*).

The third step, treatment, includes the cost of hospitalization care (fixed cost) as well as the cost of drugs and medical supplies. Preferential drug prices that are currently applicable were used in the calculations (pentamidine €1.54/vial, melarsoprol €5.3/vial), and the number of doses was 8 for pentamidine and 9 for melarsoprol. We assumed that 50% of case-patients were in stage 1 (needing pentamidine) and 50% were in stage 2 (needing melarsoprol).

### Analysis

The efficiency of each algorithm was evaluated on the basis of the cost-effectiveness ratio expressed in terms of €/life saved. This ratio was obtained by dividing the cost (in €) per person examined for each algorithm by the strategy’s effectiveness (in % HAT deaths averted per person examined), multiplied by HAT prevalence.

We used the following equations:

€/life saved = (€/person examined) / [(HAT prevalence) × (sensitivity of test sequence) × (effectiveness of HAT treatment) – (1–HAT prevalence) × (1–specificity of test sequence) × (probability of iatrogenic death)]

Cost per person examined = [(total annual operating costs of a mobile team) / (number of persons screened per year)] + [cost of confirmatory test_i_ × probability confirmatory test_i_] + [cost of treatment_k_ × probability treatment true positive)] + [cost of treatment_k_ × probability treatment false positive]

where cost of confirmatory test_i_ = cost of material + execution time + depreciation of equipment; i = LNP, FBE, TBF, CTC, mAECT, or CATT titration; cost of treatment_k_ = cost of hospitalization care + cost of drugs + cost of medical supplies; k = therapy of a true first-stage case, a true second-stage case, or a false-positive person; and probability confirmatory test_i_ = the probability of executing confirmatory test in position i in the test sequence of the evaluated algorithm. This probability was derived from the decision tree as follows:

[prevalence × sensitivity_CATT whole blood_ × (1 – sensitivity_confirmatory test 1_) × ...× (1 – sensitivity_confirmatory test i –1_)] + [(1 – prevalence) × (1 – specificity_CATT whole blood_)]

Probability of treatment of a true-positive person was calculated per stage z (1,2) as follows:

prevalence × proportion of cases__stage z__ × sensitivity__CATT whole blood__ × sensitivity__confirmatory test 1__ × ... × sensitivity__confirmatory test i–1__ × sensitivity__confirmatory test____i__

Probability of treatment of a false-positive person was generally defined as:

(1 – prevalence) × (1 – specificity__CATT whole blood__) × (1 – specificity__confirmatory test 1__) × ... × (1 – specificity__confirmatory test i–1__) × (1 – specificity__confirmatory test i__ )

However, for algorithms including CATT titration without subsequent confirmation, the probability of treatment of a false-positive person was defined as:

(1 – prevalence) × (1 – specificity__CATT whole blood__) × (1 – specificity__confirmatory test 1__) × ... × (1 – specificity__confirmatory test i–1__) × (1 – specificity__confirmatory test i__ ) + (1 – specificity__CATT whole blood__) × (1 – specificity__CATT titration__).

Effectiveness of HAT treatment was defined for true cases as:

[(proportion of stage 1 cases) × (efficacy pentamidine)] + [(proportion of stage 2 cases) × (efficacy melarsoprol)]

Finally, we calculated the incremental cost-effectiveness ratio (ICER) of saving 1 additional HAT patient by comparing each alternative algorithm to the strategy immediately above it after ranking the order of effectiveness. The ICER was calculated as:

incremental cost / (incremental effectiveness × HAT prevalence)

HAT prevalence, sensitivity, and specificity of different tests were the subject of a sensitivity analysis. A series of 1-way sensitivity analyses were conducted to examine the effect of changes in those parameters over the plausible range mentioned in Table 2 on the efficiency ranking of strategies. DATA Pro 2004 software (TreeAge, Williamstown, MA, USA) was used for this analysis.

## Results

We estimated the cost of population screening for HAT conducted by a mobile team in 2003 (excluding the cost related to confirmation and treatment) at €1.17/per person examined (Table 4). The treatment cost was estimated at €51.32/person treated with pentamidine and €129.92/person treated with melarsoprol.

**Table 4:** Cost, incremental cost, effectiveness, incremental effectiveness, cost-effectiveness, and incremental cost-effectiveness ratio of HAT screening-treatment algorithms in baseline scenario*

Algorithm	Effectiveness†	Incremental effectiveness‡	Cost per examined person, €	Incremental cost, €‡	Efficiency§	Incremental cost-effectiveness ratio¶
LNP-FBE-TBF	36.80		1.56		423.91	
LNP-CTC	55.00	18.20	1.74	0.18	316.36	98.90
LNP-CATT titration-CTC-mAECT	64.50	9.50	1.96	0.22	303.88	231.58
LNP-CTC-mAECT	77.60	13.10	2.06	0.10	265.46	76.34
LNP-CTC-CATT titration	77.80	0.20	2.82	0.76	362.47	Dominated
LNP-TBF-CTC-mAECT	79.60	2.00	2.1	0.04	263.82	200.00
LNP-TBF-CTC-mAECT-CATT titration	83.00	3.40	2.99	0.89	360.24	2617.65

*HAT prevalence 1%. HAT, human African trypanosomiasis; LNP, lymph node puncture; FBE, fresh blood examination; TBF, thick blood film; CTC, capillary tube centrifugation; CATT, card agglutination test for trypanosomiasis; CATT titration, CATT titration at end-titer 8; mAECT, mini-anion-exchange centrifugation technique. Cost/effectiveness ratio calculated as cost / (effectiveness × prevalence). Differences due to rounding in table. US $1 = Euro 0.79, Feb 2004. †% HAT deaths averted. ‡Cost and effectiveness calculated incremental to next least effective nondominated algorithm after ranking all algorithms by effectiveness. §Cost-effectiveness per life saved. ¶Incremental cost-effectiveness ratio calculated as incremental cost / (incremental effectiveness × HAT prevalence). Differences due to rounding in table. All incremental changes expressed in comparison with LNP-FBE-TBF algorithm.

Table 4 shows the cost and effectiveness per person examined for the complete screening-treatment process. Furthermore, it presents the incremental cost, the incremental effectiveness, and the incremental cost-effectiveness ratio of each algorithm compared to the next least-effective. Table 4 shows that algorithm 5 is the most cost-effective algorithm (€264.02/life saved), with algorithm 4 a close second-best option (€265.98/life saved). Although the cost per person examined for the 2 algorithms does not differ substantially, algorithm 5 is slightly more efficacious, saving 79.60% of avoidable deaths versus 77.60% for algorithm 4.

The standard algorithm (algorithm 1) had an effectiveness of 36.80% with a €/person examined of €1.56 and an efficiency of €424.94/life saved. From column 7 in Table 4, the incremental cost-effectiveness ratio to save 1 additional life ranged between €76.34 and €200/life saved for the concentration technique algorithms and €2,617.65/life saved if the decision to treat was based on serologic evidence. Algorithm 6 was dominated by algorithm 5 because algorithm 6 costs more and is less effective.

**Figure 2:**
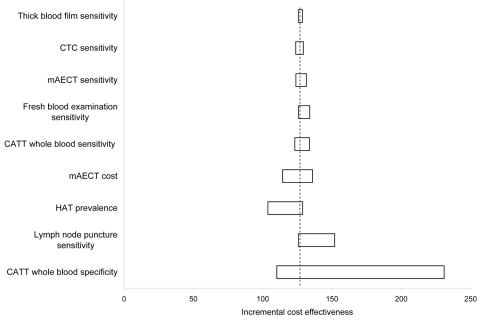
Sensitivity analysis of cost-effectiveness (€/life saved) according to variation in prevalence of human African trypanosomiasis (HAT). CTC, capillary tube centrifugation; mAECT, mini-anion-exchange centrifugation technique; CATT, card agglutination test for trypanosomiasis.

**Figure 3:**
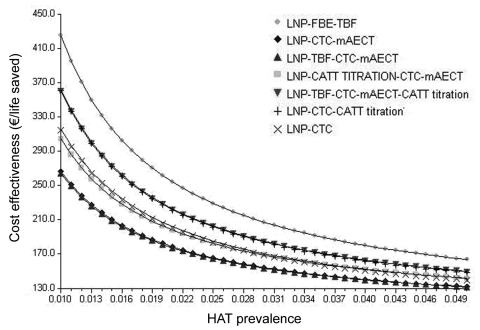
Variations in cost-effectiveness ratios as a function of prevalence of human African trypanosomiasis (HAT). LNP, lymph node puncture; FBE, fresh blood examination; TBF, thick blood film; CTC, capillary tube centrifugation; mAECT, mini-anion-exchange centrifugation technique; CATT, card agglutination test for trypanosomiasis; CATT titration, CATT titration at end-titer 8.

The sensitivity analysis showed that our conclusion remained robust to variation over the range of uncertainty in all parameters included in the model (Figure 2). Changes in the specificity of CATT and CATT titration, cost of pentamidine, and HAT prevalence decreased the difference in the cost-effectiveness ratio with the next most efficient algorithm. Figure 3 shows the variation of the cost-effectiveness ratio in function of the prevalence.

## Discussion

Current observations suggest that parasitologic confirmation tests of inadequate sensitivity lead to suboptimal effectiveness of HAT active case-finding programs (*4*). Our analysis shows that concentration techniques, and to a lesser extent, serologic tests can substantially improve the efficiency of HAT confirmation algorithms. The currently used algorithm (standard algorithm) was the least cost-effective of all those compared***,*** mainly because its sensitivity is so low. Low effectiveness of the standard algorithm was also reported by Paquet et al. (*5*), Pépin et al. (*6*), and recently by Robays et al. (*13*) who put it respectively at 39.5%, 20–30%, and 50%. The greater effectiveness of concentration techniques (currently €2.82/test for mAECT) more than compensates for the higher cost. Serologic algorithms were not more cost-effective than the algorithms that included concentration techniques, but the difference in efficiency decreased at a higher prevalence (Figure 3). Algorithms combining CATT titration with subsequent confirmation by concentration techniques were not competitive in this analysis; efficiency always remained lower than that of algorithms based exclusively on concentration techniques. This is due to the loss of patients caused by the suboptimal sensitivity of CATT titration.

Our study could shed some light on the controversial issue of treating for HAT on the basis of serologic evidence. In a similar discussion in the field of kala-azar case management, Boelaert et al. (*14*) argued that a serologic algorithm was more cost-effective, and therefore a better choice, than a parasitologic algorithm with poor sensitivity.

In this study, we also find that treating patients suspected by serologic tests to have HAT is a better option than using the current algorithm without concentration techniques. This may be a valuable strategy when introduction of mAECT or CTC is not yet feasible. A limitation of the study is that the specificity values of CATT and CATT dilution are based on data from 1 region and might be underestimated given findings from routine data from the national program (*4*). However, this regional variation remains poorly documented, and better estimations of the specificity of CATT in different regions are needed. The use of CATT titration has primarily been evaluated in the group of CATT whole blood–positive persons who were negative for parasites. Chappuis et al. (*15*), Simarro et al. (*16*), Van Nieuwenhove and Declercq (*17*), Frézil et al. (*18*), and Bruneel et al*.* (*19*) evaluated the strategy of treating persons who had positive serologic test results (indirect immunofluorescent antibody test or CATT) and who were negative for parasites and found this strategy more effective than that based on parasitologic confirmation.

Our model did not give different weights to iatrogenic deaths of noninfected persons compared with infected persons. This point should be carefully considered because the serologic algorithm will expose some noninfected persons to toxic drugs. However, as no treatment with second-stage HAT drugs will be given unless a lumbar puncture and parasitologic confirmation in cerebrospinal fluid are obtained, the exposure of noninfected persons to the highly toxic second-stage HAT drugs will be minimal, even under a serologic algorithm.

Our decision analysis is based on a model that depends on certain assumptions made for the purpose of simplification. An important assumption is that a true HAT case missed by the mobile teams will eventually die; in practice, if treatment is sought, this patient’s condition might be diagnosed and cured, or alternatively, HAT could be detected later by the mobile team on a second visit. We examined whether this assumption would change our conclusions by hypothesizing that 40% of such HAT case-patients would be detected and treated at a later stage. In this scenario, the differences between algorithms in terms of cost-effectiveness are reduced without altering the relative order (data not shown).

Second, confirmation tests in the sequence were used as if they were independent, but in reality, this is unlikely. For example, mAECT sensitivity could be different in a group of TBF-positive persons compared to a group of TBF-negative persons. We have examined the effect of this conditional relationship on the findings of our study by means of sensitivity analysis (data not shown). Once again, these variations did not affect the ranking of the results.

The cost of the mobile team per person screened was estimated on the basis of 40,000 examinations per year (*20*). Even if the program could screen 60,000 persons per mobile team per year, it would not change the rank order of efficiency. Finally, better confirmation algorithms for HAT may also have a beneficial effect on transmission because there will be fewer undetected cases to spread the disease in the community. If such an effect is considered, it might favor the efficiency of treatment based on serologic markers, which may lead to a faster reduction of the human reservoir similar to the chemoprophylaxis campaigns of the 1950s. Unfortunately, no sufficiently validated models for *T. b. gambiense* sleeping sickness transmission allow for the estimation of this potential benefit at population level. Our analysis disregarded this potential future benefit.

A policy change in HAT population screening seems definitely needed, and there is ample scope for improving the sensitivity of the confirmation stage. Introducing algorithm 5 has an incremental cost-effectiveness ratio of €200.00/(additional) life saved. This ratio represents the cost to HAT control programs of shifting to algorithm 5 (the most cost-effective) to save an additional life. This choice seems very rational. The incremental cost-effectiveness ratio was €76.34 if HAT control programs chose algorithm 4. TBF is a lengthy procedure, and dropping it from the sequence has logistic and organizational advantages. Our calculations were based on an estimate of 47 minutes of staff time required for TBF, obtained in a previous study (*4*). However, because labor costs are so low in the DRC and TBF does not require expensive reagents or equipment, it remains a very affordable test, whenever there are no time constraints for staff.

In conclusion, the standard HAT screening algorithm has low sensitivity and is inefficient. Inclusion of concentration techniques in HAT screening algorithms can be recommended as cost-effective alternatives. The use of serologic algorithms should be studied further before being recommended for HAT population screening.
